# Optimizing Red Light-Based Photodynamic Therapy for Effective Bactericidal Action Against *Fusobacterium nucleatum* Subspecies

**DOI:** 10.3390/pathogens13111016

**Published:** 2024-11-19

**Authors:** Jianglan Li, Takayuki Nambu, Chao Wang, Hiroki Takigawa, Hugo Maruyama, Chiho Mashimo, Toshinori Okinaga

**Affiliations:** 1 Graduate School of Dentistry (Bacteriology), Osaka Dental University, Hirakata 573-1121, Japan; li-j@cc.osaka-dent.ac.jp; 2 Department of Microbiology, Osaka Dental University, Hirakata 573-1121, Japan; takigawa@cc.osaka-dent.ac.jp (H.T.); maruyama@cc.osaka-dent.ac.jp (H.M.); mashimo@cc.osaka-dent.ac.jp (C.M.); 3 Center of Oral Implantology, Stomatological Hospital, School of Stomatology, Southern Medical University, Guangzhou 510515, China; wangchao101@smu.edu.cn

**Keywords:** *Fusobacterium nucleatum*, photodynamic therapy, 5-aminolevulinic acid, subspecies, red light-emitting diode

## Abstract

*Fusobacterium nucleatum* (*F. nucleatum*), a key pathogen implicated in periodontal disease, contributes to oral biofilm maturation and is linked to development of systemic diseases like colorectal cancer and liver cirrhosis. Photodynamic therapy (PDT) combined with 5-aminolevulinic acid (5-ALA) treatment (ALA-PDT) selectively targets *F. nucleatum* by inducing porphyrin accumulation. The bactericidal effect of red light-based PDT on *F. nucleatum* has not been evaluated previously. This study investigates the effect of ALA-PDT using red light-emitting diode (LED) light on *F. nucleatum* subspecies and their porphyrin accumulation. *F. nucleatum* subspecies were cultured with varying concentrations of 5-ALA under anaerobic conditions. Porphyrin accumulation was measured via fluorescence spectroscopy, and colony-forming units were measured to determine bacterial viability post-treatment. Additionally, other subspecies responded well to 0.01% 5-ALA, and uroporphyrin I accumulation correlated with bacterial death, revealing optimal bactericidal conditions. These results suggest that optimizing light intensity and 5-ALA concentration can significantly enhance the therapeutic potential of ALA-PDT in oral healthcare.

## 1. Introduction

Periodontal disease is characterized by chronic inflammation of the tissues surrounding the teeth, leading to alveolar bone resorption and ultimately tooth loss [1]. In addition to impairing oral function, periodontal disease has been linked to the development of various systemic diseases such as cardiovascular disease, diabetes, aspiration pneumonia, and chronic obstructive pulmonary disease [2].

*Fusobacterium nucleatum* (*F*. *nucleatum*), a Gram-negative anaerobic bacterium commonly found in the oral cavity, represents one of the causative agents of periodontal disease [3]. This bacterium establishes itself after the initial colonization of dental plaque, and it plays a crucial role in the maturation of oral biofilms by facilitating the adhesion and growth of late-colonizing bacteria [4]. Furthermore, *F. nucleatum* has been implicated in the development of several systemic diseases, including colorectal cancer, liver cirrhosis, inflammatory bowel disease, and Alzheimer’s disease [5,6]. Therefore, the development of methods to efficiently control the abundance of *F. nucleatum* in oral biofilms is of great importance.

We recently reported that photodynamic therapy combined with 5-aminolevulinic acid (5-ALA) treatment (ALA-PDT) can selectively eradicate *F. nucleatum* in vitro, even in the presence of other oral bacteria [7]. When taken up by bacteria, the amino acid 5-ALA is metabolized to produce porphyrins, which accumulate intracellularly. These porphyrins are excited by light at specific wavelengths, particularly blue (405 nm) and red (635 nm) light, resulting in singlet oxygen generation, which induces cell damage [8]. While ALA-PDT exhibits bactericidal activity against pathogens, such as methicillin-resistant *Staphylococcus aureus* and *Mycobacterium marinum* [9,10], it is believed to have a lower likelihood of inducing treatment resistance compared to that induced by conventional antibiotics [11]. In the case of *F. nucleatum*, 5-ALA supplementation leads to the accumulation of uroporphyrin I, which is thought to be responsible for the selective killing of *F. nucleatum* in mixed oral bacterial populations. Previous studies demonstrated the bactericidal effect using blue (405 nm) LED light; however, its limited tissue penetration has restricted its clinical use. In contrast, red light (635 nm) is more commonly used in clinical settings, and it can penetrate more than 2 mm of mucosal tissues [12], making it more likely to reach bacteria within the subgingival plaque. *F. nucleatum* comprises four subspecies, namely, *animalis*, *nucleatum*, *vincentii*, and *polymorphum*, with some evidence suggesting that they may represent distinct species [13]. These subspecies vary significantly in their association with disease and biofilm formation capabilities [14,15], thereby highlighting the need for subspecies-level analysis. Developing a bactericidal method effective against all *F. nucleatum* subspecies is essential for the more precise control of subgingival biofilm formation. However, studies investigating the bactericidal effects of ALA-PDT on *F. nucleatum* subspecies remain limited.

In this study, we aimed to evaluate the efficiency of ALA-PDT using red LED light in the eradication of *F. nucleatum* in vitro, and we aimed to investigate differences in porphyrin accumulation and bactericidal effects at the subspecies level of *F. nucleatum*.

## 2. Materials and Method

### 2.1. Materials

5-ALA (phosphate salt) was obtained from SBI Pharmaceuticals (Tokyo, Japan). Stock solutions of 5-ALA were prepared by dissolving 5-ALA in sterile distilled water to a final concentration of 50 mg/mL and filtering through a 0.20 µm membrane (ADVANTEC, Tokyo, Japan). The solution was stored in the dark at 4 °C. Prior to each experiment, the pH of the 5-ALA solution was adjusted to 7 using 1 M sodium hydroxide (NaOH).

### 2.2. Bacterial Strains and Growth Conditions

The bacterial strains used in this study included *F. nucleatum* subsp. *animalis* (Fna) JCM11025, *F. nucleatum* subsp. *polymorphum* (Fnp) JCM12990, *F. nucleatum* subsp. *vincentii* (Fnv) JCM11023, and *F. nucleatum* subsp. *nucleatum* (Fnn) JCM8532. These strains were cultured anaerobically in Gifu anaerobic medium (mGAM) (Nissui Pharmaceutical, Tokyo, Japan) for 24 h at 37 °C under an atmosphere comprising 80% nitrogen, 10% hydrogen, and 10% carbon dioxide.

### 2.3. Fluorescence Spectroscopy Measurements

Bacterial cultures were treated with 5-ALA by mixing 5-ALA (pH adjusted to 7 with 1 M NaOH), 2 mL mGAM broth, phosphate-buffered saline (PBS, pH 7.4; Nippon Gene, Tokyo, Japan), and bacterial cells adjusted to an optical density (OD) of 0.1 at 600 nm using a ColourWave CO7500 colorimeter (WPA, Holliston, MA, USA) [7]. The 5-ALA was added to obtain final concentrations ranging from 0.001 to 0.5% (*w*/*v*). The samples were incubated in the dark at 37 °C with shaking for 20 h. Following incubation, 2 mL of the bacterial suspension was centrifuged (13,200 rpm for 4 min; Eppendorf, Minispin, Hamburg, Germany), and the pellet was resuspended in 500 µL of 6 M formic acid. The samples were incubated in the dark at 37 °C for 30 min in a Bio-shaker (BR-300 LF; TAITEC, Saitama, Japan). After centrifugation at 13,200 rpm for 4 min, the supernatant was collected and transferred to a 96-well plate (100 µL per well, with each sample in triplicate). Fluorescence spectra were measured using a SpectraMax M5 microplate reader (Molecular Devices, San Jose, CA, USA), with excitation at 405 nm and emission recorded between 550 and 700 nm. Measurements were taken at intervals of 2 nm for emission paths.

### 2.4. Light Source

A red LED (wavelength: 635 nm; Aladuck GR, SBI Pharmaceuticals Co., Ltd.) and a blue LED (wavelength: 400–410 nm; Aladuck LS-DLED, SBI Pharmaceuticals Co., Ltd.) were used. LED output power was calibrated to a constant intensity of 0.050 W/cm^2^ for the red LED and 0.162 W/cm^2^ for the blue LED using a light power meter (Astral AI310; Scientech Inc., Boulder, CO, USA) prior to each experiment. The lamp was positioned above the 12-well plate (Falcon; Corning Inc., Corning, NY, USA) at a distance of 4.5 cm for irradiation.

### 2.5. Photodynamic Inactivation of F. nucleatum Subspecies

*F. nucleatum* subspecies were anaerobically cultured overnight at 37 °C in mGAM. Bacterial cells were centrifuged at 13,200 rpm for 4 min (Eppendorf Minispin), and the pellet was resuspended in PBS and adjusted to an OD600 of 0.1. A final volume of 2.5 mL was prepared by mixing 5-ALA (pH adjusted to 7 with 1 M NaOH), 2 mL of mGAM broth, PBS, and the bacterial cell suspension. The mixture was incubated at 37 °C for 20 h [7]. After incubation, the bacterial pellet was harvested, resuspended in PBS, and diluted to an OD600 of 0.4. A total of 1 mL of the bacterial suspension was added to each well of a 12-well plate. LED irradiation was performed at light doses of 20 J/cm^2^, 40 J/cm^2^, and 80 J/cm^2^ by increasing the exposure time (400, 800, and 1600 s, respectively). Serial dilutions were plated on mGAM agar plates, and the colony-forming units (CFU/mL) were counted after 48 h of incubation.

### 2.6. Statistical Analysis

All experiments were conducted in triplicate. Differences between groups were analyzed using Student’s *t*-test in GraphPad Prism 10.0 (GraphPad Software, San Diego, CA, USA). Data are presented as the mean ± standard deviation, and a *p*-value of <0.05 was considered significant.

## 3. Results

### 3.1. Bactericidal Effect of Red LED-Based ALA-PDT on Fusobacterium

We previously observed a significant bactericidal effect when Fnp was cultured with 0.5% 5-ALA and exposed to blue LED light irradiation at an intensity of 18 J/cm^2^ [7]. When the same intensity of red LED light (18 J/cm^2^) was applied to Fnp cultured with 0.5% 5-ALA for 20 h, a slight reduction in CFU was observed, but it was not significant (Figure S1). However, when the irradiation intensity was increased to 40 J/cm^2^, a significant bactericidal effect (1.5 log reduction) was observed. Moreover, this CFU reduction was not observed under conditions with either 5-ALA treatment or LED irradiation alone. These findings indicate that although a relatively higher irradiation intensity is required, red LED-based ALA-PDT can achieve bactericidal effects against Fnp, similar to blue LED-based treatment.

### 3.2. Production of Uroporphyrin I and Bactericidal Effect at Different 5-ALA Concentrations

**Figure 1:**
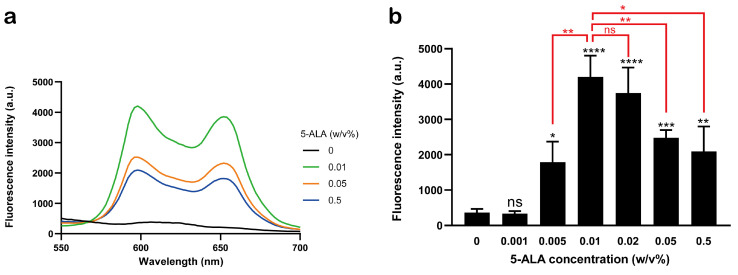
Porphyrin synthesis in *F. nucleatum* subsp. *polymorphum* treated with different 5-ALA concentrations. (**a**) Fluorescence emission spectra after 20 h of incubation in media containing different concentrations of 5-ALA. (**b**) Fluorescence intensity at 598 nm after incubation with various 5-ALA concentrations (*n* = 3). ns, no significance. * *p* < 0.05, ** *p* < 0.01, *** *p* < 0.001, and **** *p* < 0.0001, compared with sham treatment or between indicated groups. Student’s *t*-test. Data are presented as the mean ± standard deviation. 5-ALA, 5-aminolevulinic acid.

**Figure 2:**
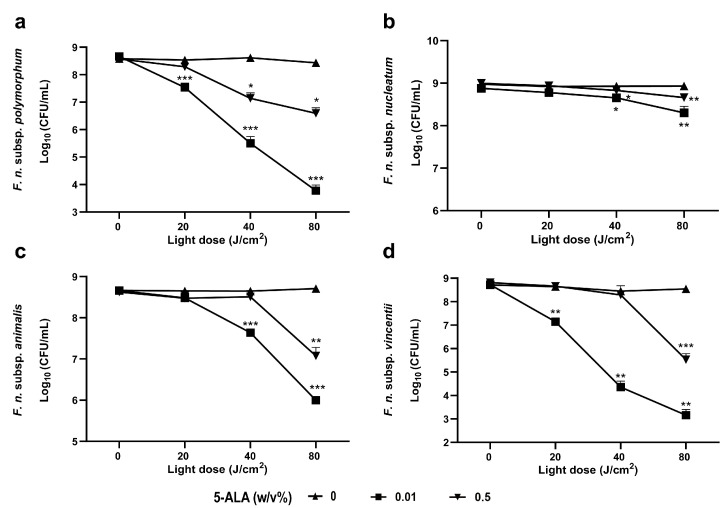
Bactericidal effect of 5-ALA-photodynamic therapy on subspecies of *F. nucleatum*. *F. nucleatum* subspecies (*polymorphum* (**a**), *nucleatum* (**b**), *animalis* (**c**), and *vincentii* (**d**)) were cultured with 0.01% or 0.5% 5-ALA and exposed to red LED light at doses ranging from 0 to 80 J/cm^2^ (*n* = 3). * *p* < 0.05, ** *p* < 0.01, and *** *p* < 0.001, compared with sham treatment. Student’s *t*-test. Data are presented as the mean ± standard deviation from three independent experiments. 5-ALA, 5-aminolevulinic acid.

To further optimize the conditions for inducing the bactericidal effect of red LED-based ALA-PDT on Fnp, we evaluated the effect of different 5-ALA concentrations. We previously demonstrated that uroporphyrin I, which is involved in singlet oxygen generation, accumulates within bacterial cells when Fnp is cultured with 5-ALA [7]. Therefore, Fnp was cultured for 20 h with 0–0.5% 5-ALA, and uroporphyrin I accumulation was measured via fluorescence spectroscopy. The characteristic fluorescence spectrum of uroporphyrin I was not detected in the absence of 5-ALA treatment; however, significant fluorescence was observed even at lower concentrations of 5-ALA treatment, including 0.01% (Figure 1a). A detailed analysis at the fluorescence peak wavelength (598 nm) revealed that 0.01% 5-ALA treatment resulted in approximately twice the fluorescence intensity of 0.5% (Figure 1b). Additionally, the fluorescence intensity increased with incubation time after adding 0.01% 5-ALA (Figure S2). We next investigated whether the variation in uroporphyrin I production associated with different 5-ALA concentrations was related to the bactericidal effect of ALA-PDT. Fnp was cultured with 5-ALA and exposed to 20 J/cm^2^ red LED irradiation. No significant bactericidal effect was observed with 0.5% 5-ALA treatment; however, a significant 1 log reduction in CFU was observed when cultured with 0.01% 5-ALA (Figure 2a). Furthermore, more than a 4 log reduction in CFU was observed at an irradiation intensity of 80 J/cm^2^. These results indicate that treating Fnp with 0.01% 5-ALA provides a sufficient bactericidal effect with an irradiation intensity of 20 J/cm^2^.

To elucidate the mechanism by which the uroporphyrin I production and bactericidal activity of PDT are maximized when Fnp is cultured with 0.01% 5-ALA, we analyzed the expression changes in genes involved in uroporphyrin I synthesis from 5-ALA. RNA was extracted from Fnp cells during culture, and reverse transcription–polymerase chain reaction assays were performed to assess changes in gene expression (the specific primers are listed in Table S1). No significant changes in gene expression were observed after treatment with either 0.5% or 0.01% 5-ALA (Figure S3). Additionally, scanning electron microscopy analysis revealed no morphological changes in the cells caused by 5-ALA supplementation during culture (Figure S4).

### 3.3. Effect of ALA-PDT Treatment on Other Subspecies of F. nucleatum

Next, we assessed the bactericidal effect of ALA-PDT on three other subspecies (Fna, Fnn, and Fnv). Similarly to Fnp, each subspecies was cultured with different concentrations of 5-ALA and exposed to red LED irradiation at intensities of 20, 40, and 80 J/cm^2^. In all subspecies, survival was not reduced by LED irradiation alone at doses up to 80 J/cm^2^ (Figure 2b–d). However, bactericidal effects were observed after LED irradiation combined with 5-ALA treatment in all subspecies, with a greater effect observed using 0.01% 5-ALA compared to that obtained using 0.5%. At 0.5% 5-ALA treatment, only minimal or no significant bactericidal effect was observed after 40 J/cm^2^ irradiation, whereas a significant reduction in CFU was observed at 0.01% 5-ALA treatment. Further, the extent of bactericidal activity varied between subspecies, with Fnp and Fnv showing relatively greater susceptibility to ALA-PDT treatment, Fna showing moderate susceptibility, and Fnn showing little to no reduction in CFU even at 80 J/cm^2^ irradiation.

**Figure 3:**
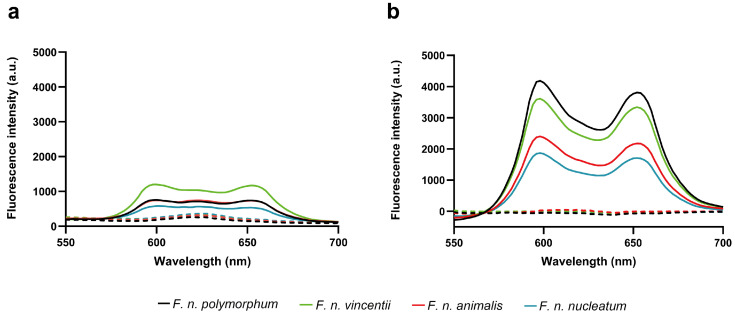
Fluorescence emission spectra of *F. nucleatum* subspecies. Fluorescence spectra of *F. nucleatum* subspecies after 20 h of incubation with or without 0.5% (**a**) or 0.01% (**b**) 5-ALA were measured using fluorescence spectroscopy. Solid lines represent the addition of 5-ALA, and dotted lines indicate the absence of 5-ALA. 5-ALA, 5-aminolevulinic acid.

**Figure 4:**
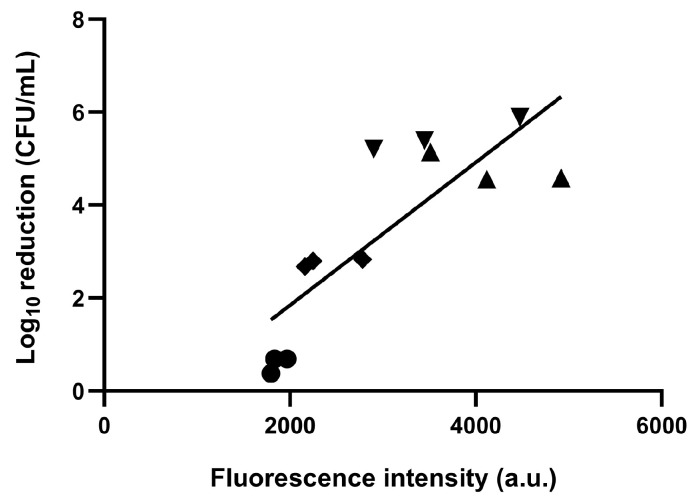
Correlation between the bactericidal effect of 5-aminolevulinic acid-photodynamic therapy on *F. nucleatum* subspecies and the amount of porphyrin produced. *F. nucleatum* subspecies: filled circles (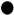): *nucleatum*; filled rhombuses (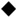): *animalis*; filled equilateral triangles (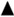): *polymorphum*; filled inverted triangles (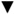): *vincentii*.

To evaluate the differences in bactericidal effects among subspecies, we measured the accumulation of uroporphyrin I in each subspecies after treatment with 0.01% and 0.5% 5-ALA and culturing for 20 h. Fluorescence intensity was detected using fluorescence spectroscopy in all subspecies under 5-ALA treatment conditions (Figure 3), and the intensity was greater in the 0.01% 5-ALA group than in the 0.5% 5-ALA group. Spearman’s correlation analysis was performed between the fluorescence intensity at the 598 nm peak and the reduction in CFU by ALA-PDT. A significant and strong positive correlation was observed between the fluorescence intensity of porphyrins corresponding to uroporphyrin I and the bactericidal effect of ALA-PDT (Spearman’s ρ = 0.844; *p* = 0.001; Figure 4).

## 4. Discussion

In this study, we demonstrated that *F. nucleatum* can be eradicated via ALA-PDT using a 635 nm red LED. We previously showed that ALA-PDT using 405 nm blue LED irradiation at 18 J/cm^2^ exerts a bactericidal effect on Fnp [7]. In contrast, under the same light energy dose of 18 J/cm^2^ with 0.5% 5-ALA and a 635 nm red LED, no significant bactericidal effect was observed. A bactericidal effect can only be achieved when the irradiation intensity of the red LED increases to 40 J/cm^2^. This suggests that red light may require a higher irradiation intensity than blue LED to achieve a similar bactericidal effect in ALA-based photodynamic therapy. However, since the red LED source used in this study had a lower output (0.050 W/cm^2^ compared to 0.162 W/cm^2^), further analysis is needed to gain deeper insights, including investigations into reactive oxygen species generation and absorption, as well as cell structure repair mechanisms. These findings are consistent with findings on *Propionibacterium acnes* [16]. Owing to the specific properties of porphyrins, 405 nm light is optimal for porphyrin excitation [17]. Although red light at 635 nm requires a relatively higher intensity to achieve a bactericidal effect, its deeper penetration in biofilms and tissues suggests that its use may be more advantageous for targeting the subgingival plaque and periodontal tissues.

The addition of 5-ALA to culture media results in the accumulation of specific porphyrins within microbial cells across various species. In our previous research, high-performance liquid chromatography analysis revealed the accumulation of uroporphyrin I in 5-ALA-treated Fnp [7]. In the present study, uroporphyrin I peaks were detected in all subspecies of *F. nucleatum* when cultured with 5-ALA. However, higher levels of uroporphyrin I were accumulated in cells treated with 0.01% 5-ALA than those in 0.5% 5-ALA-treated cells. This suggests that the accumulation of endogenous porphyrins in Fnp is not entirely dependent on 5-ALA concentration. Similar phenomena have been observed in *Staphylococcus epidermidis* and *Candida albicans*, where *Staphylococcus epidermidis* predominantly synthesizes uroporphyrin III, while *Candida albicans* primarily produces protoporphyrin IX [18,19]. Further, the lack of correlation between ALA concentration and porphyrin accumulation has been observed with different types of porphyrins. Three metabolic pathways for 5-ALA have been determined [20], which produce various porphyrins such as uroporphyrin I, uroporphyrin III, coproporphyrin, and protoporphyrin IX. The enzymes involved in these common metabolic pathways for 5-ALA include HemB, HemC, and HemD, which significantly influence the production of these porphyrins. For instance, overexpressing HemB, HemC, and HemD to promote the uroporphyrinogen III synthesis pathway increases heme production by 39% in *Bacillus subtilis* [21]. We hypothesized that 5-ALA concentration may influence the expression of HemB, HemC, and HemD, thus affecting porphyrin production. To test this hypothesis, we cultured Fnp together with 0.01% and 0.5% 5-ALA and compared the expression levels of *hemB*, *hemC*, and *hemD* (Figure S3). Furthermore, we observed Fnp morphology using scanning electron microscopy (Figure S4). However, no changes in gene expression or morphology were detected in relation to 5-ALA concentration, and we could not clarify the relationship with uroporphyrin I accumulation. In *Escherichia coli* K-12, a relationship between the 5-ALA-induced formation of endogenous porphyrins and the bactericidal effect of ALA-PDT has been reported [22]. To further enhance the bactericidal effect in *F. nucleatum*, it is necessary to conduct a more detailed analysis of the mechanism underlying uroporphyrin I accumulation.

We found that the bactericidal effect of ALA-PDT varied significantly among the subspecies of *F. nucleatum*. Fnp and Fnv were highly sensitive to ALA-PDT, Fna showed moderate sensitivity, and Fnn exhibited low sensitivity. Additionally, this sensitivity correlated with the amount of uroporphyrin I accumulated. Previous studies have reported that the four subspecies differ in their biofilm-forming ability and pathogenicity [15,23], and our results showed significant differences in uroporphyrin I accumulation. Despite the promising findings of this study, several limitations should be acknowledged. First, the experiments were conducted in vitro, and while these conditions provide valuable insights, they do not fully replicate the complex environment of the oral cavity in vivo. Factors such as saliva flow, immune responses, and microbial interactions within a live host may influence the effectiveness of ALA-based photodynamic therapy. Secondly, in this study, the bacterial cells exposed to LED irradiation were in the stationary phase, and bacteria at different growth stages may exhibit varying sensitivities to ALA-based photodynamic therapy. In particular, within biofilms, a diverse community of bacterial species exists at different growth stages, necessitating the consideration of factors such as 5-ALA penetration into cells and the exchange of metabolites. Additionally, the study focused on a limited number of *F. nucleatum* subspecies, and further research is required to evaluate the bactericidal efficacy of ALA-based photodynamic therapy across a broader range of clinically relevant subgroups and strains. Furthermore, it should be considered that porphyrins produced by *F. nucleatum* or other oral bacterial species may be secreted within the biofilm, potentially exerting bactericidal effects on other bacterial species. Although red LED light with high penetration power has been shown to be effective against *F. nucleatum*, the clinical effectiveness of this method needs to be tested in real-world periodontal treatment settings. Finally, the long-term effects of repeated ALA-based photodynamic therapy treatments on both pathogenic and beneficial oral microbiota were not investigated, which could have implications for the overall balance of the oral microbiome. These metabolic differences among subspecies suggest that more detailed analysis at the subspecies level is needed to further understand the characteristics of *F. nucleatum*.

## 5. Conclusions

ALA-PDT was found to exhibit effective bactericidal activity against *F. nucleatum* when using red light irradiation. The optimal bactericidal conditions for ALA-PDT were identified, showing that a 5-ALA concentration of 0.01% yielded the most effective results, with varying bactericidal effects observed across subspecies. The bactericidal efficacy of ALA-PDT among different subspecies of *F. nucleatum* was positively correlated with the fluorescence intensity of the porphyrins produced.

## Data Availability

The original contributions presented in the study are included in the article/Supplementary Materials, and further inquiries can be directed to the corresponding authors.

## Supplementary Materials

The following supporting information can be downloaded at: https://www.mdpi.com/article/10.3390/pathogens13111016/s1, Figure S1: Bactericidal effect of photodynamic therapy using 5-ALA on *F. nucleatum* subsp. *polymorphum*; Figure S2: Porphyrin synthesis in *F. nucleatum* subsp. *polymorphum* with 0.01% 5-aminolevulinic acid under different incubation times; Figure S3: Real-time reverse transcription–polymerase chain reaction analysis evaluated the relative expression levels of *hemB*, *hemC*, and *hemD* in *F. nucleatum* subsp. *polymorphum*; Figure S4: Scanning electron microscopy images of *F. nucleatum* subsp. *polymorphum* after incubation with different concentrations of 5-ALA for 20 h. Table S1: Primers for real-time reverse transcription–polymerase chain reaction analysis of *F. nucleatum* subsp. *polymorphum*.

## Abbreviations

5-ALA	5-aminolevulinic acid
PDT	photodynamic therapy
LED	light-emitting diode
*Fusobacterium nucleatum*	*F* *. nucleatum*
Fna	*F. nucleatum* subsp. *Animalis*
Fnp	*F. nucleatum* subsp. *Polymorphum*
Fnv	*F. nucleatum* subsp. *Vincenti*
Fnn	*F. nucleatum* subsp. *Nucleatum*
mGAM	Gifu anaerobic medium
