# Supplementary material for: Optimizing Red Light-Based Photodynamic Therapy for Effective Bactericidal Action Against Fusobacterium nucleatum Subspecies
Source: Pathogens. 2024 Nov 19;13(11):1016. doi: 10.3390/pathogens13111016 (PMC11597717; doi:10.3390/pathogens13111016)
Supplement: Supplementary file 1 [file pathogens-13-01016-s001.zip › pathogens-3286513-supplementary.pdf]

Figure S1

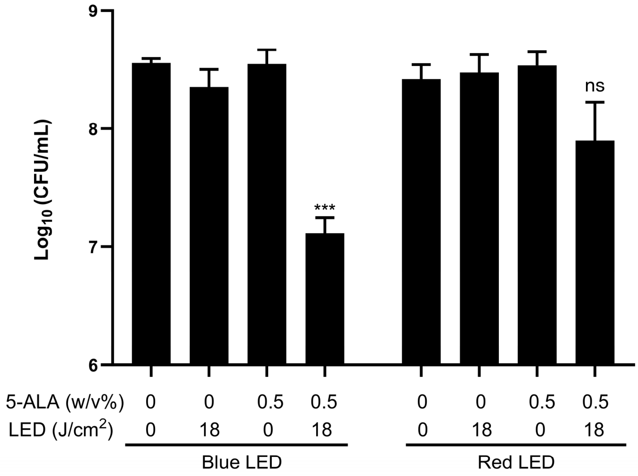

Figure S1. Bactericidal effect of photodynamic therapy using 5-ALA on *Fusobacterium nucleatum* subsp. *polymorphum*. The changes in CFU with or without the addition of 0.5% 5-ALA and exposure to blue (400 nm) or red (635 nm) LED irradiation are shown (n = 3). ***$p < 0.001$ vs. respective controls; ns, no significance; Student's t-test. Data are presented as the mean ± standard deviation. 5-ALA, 5-aminolevulinic acid; CFU, colony-forming units; LED, light-emitting diode.

Figure S2

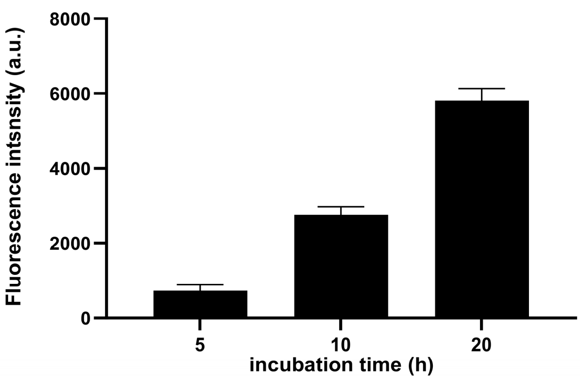

Figure S2. Porphyrin synthesis in *Fusobacterium nucleatum* subsp. *polymorphum* with 0.01% 5-aminolevulinic acid under different incubation times (n = 3).

Figure S3

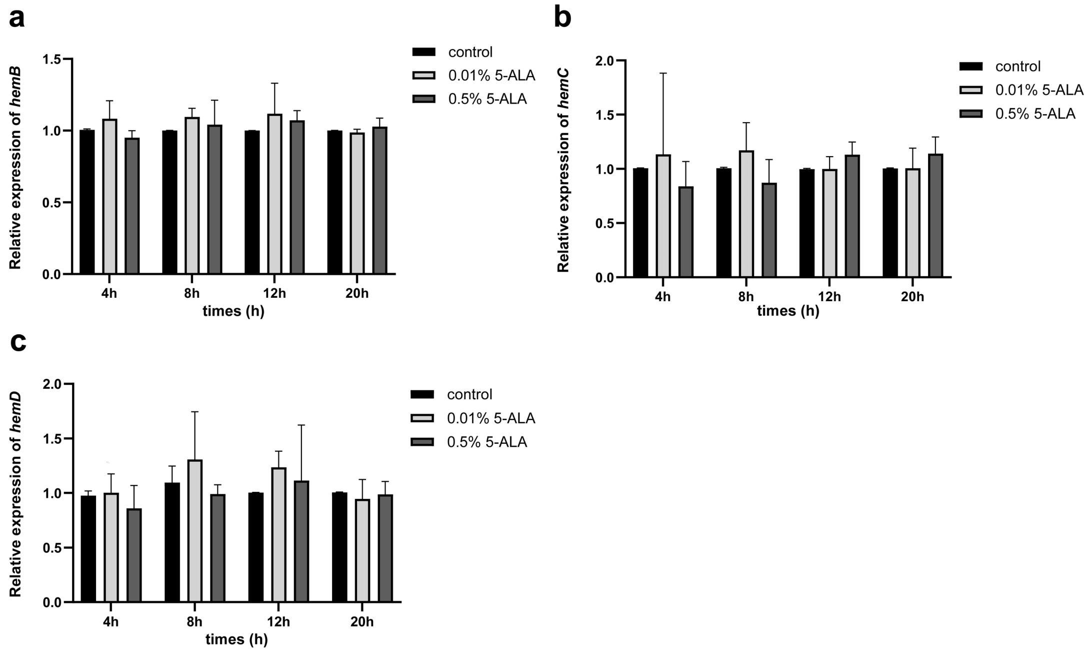

Figure S3. Real-time reverse transcription–polymerase chain reaction analysis evaluated the relative expression levels of *hemB, hemC,* and *hemD* in *Fusobacterium nucleatum* subsp. *polymorphum*. The expression level of each gene in the bacterial cultures treated with 0.01% and 0.5% 5-ALA was compared to that of control bacteria without 5-ALA treatment after 4, 8, 12, and 20 h. Expression levels of (a) *hemB*, (b) *hemC*, and (c) *hemD* are shown (n = 3). 5-ALA, 5-aminolevulinic acid.

Methods

Quantitative reverse transcription–polymerase chain reaction (qRT-PCR) was conducted to confirm the expression levels of the *hemB*, *hemC*, and *hemD* genes in *F. nucleatum* subsp. *polymorphum*. The *rpoB* gene was used as a reference gene. *F. nucleatum* subsp. *polymorphum* was grown overnight, the culture was adjusted to an OD_{600} of 0.1, and 0.01% or 0.5% 5-aminolevulinic acid (5-ALA) was added to the

cultures for 4, 8, 12, and 20 h at 37 °C under anaerobic conditions. Bacteria cultured without 5-ALA served as the control group. Bacterial cells were centrifuged at 13,200 rpm for 5 min, the supernatant was removed, and the cells were adjusted to an OD_{600} of 1. Total mRNA was isolated and purified using the RNeasy Mini Kit (Qiagen, San Diego, CA, USA). cDNA was synthesized using the ReverTra Ace® qPCR RT Master Mix (TOYOBO, Osaka, Japan). qRT-PCR was conducted using THUNDERBIRD® SYBR™ qPCR Mix (TOYOBO) on the QuantStudio™ 3 Real-Time PCR System (Thermo Fisher Scientific, Waltham, MA, USA). The thermal cycling conditions were as follows: 95°C for 60 s, followed by 40 cycles of 95°C for 5 s, 55°C for 20 s, and 72°C for 20 s. Relative gene expression levels were calculated using the $2^{-\Delta\Delta Ct}$ method.

Table S1. Primers for real-time reverse transcription–polymerase chain reaction analysis of *Fusobacterium nucleatum* subsp. *polymorphum*

Genes	Primer sequence
rpoB	Forward 5′-ACAGAACCACCTGCTGCTGAAGA-3′<br>Reverse 5′-ACCCCTTTGTTTCCGTGTCT-3′
hemB	Forward 5′-TGAGATAGGAAGCCAAGCCT-3′<br>Reverse 5′-GCTCCTGCTTTTGCATGAGAT-3′
hemC	Forward 5′-GGCTCTTGCTCAAGCGAATC-3′<br>Reverse 5′-CCCTTAGGTGATATAGCAGGCA-3′
hemD	Forward 5′-GGCAGGACCAGGAGATTTTG-3′<br>Reverse 5′-GGATCTCCACCTTTTACTCTAGC-3′

Figure S4

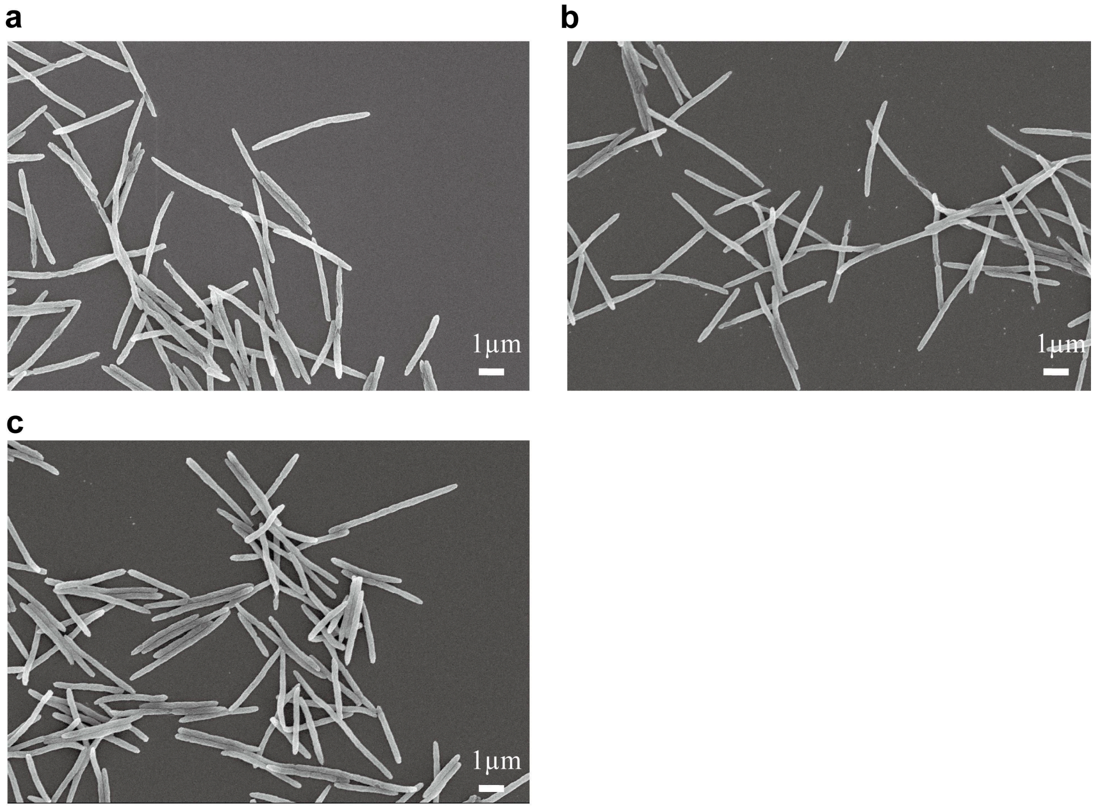

Figure S4. Scanning electron microscopy images of *Fusobacterium nucleatum* subsp. *polymorphum* after incubation with different concentrations of 5-ALA for 20 h. Images of cells treated with (a) 0% 5-ALA, (b) 0.01% 5-ALA, and (c) 0.5% 5-ALA are shown. 5-ALA, 5-aminolevulinic acid.

Methods

F. nucleatum subsp. *polymorphum* cultures were treated with 0.01% and 0.5% 5-ALA and incubated anaerobically at 37°C for 20 h. Bacterial suspensions were then centrifuged at 5,000 rpm for 4 min and dehydrated using a graded ethanol series (50%, 60%, 70%, 80%, 90%, and 99.5% v/v), with each step lasting 15 min. The bacterial cells were subsequently dropped onto glass coverslips and fixed with 500 µl of

tert-butyl alcohol for 1 h, followed by freeze-drying. The samples were sputter-coated with osmium before imaging using a Hitachi S-4800 scanning electron microscope (Hitachi, Tokyo, Japan) at 5 kV.
